# Early-Life Slow Enteral Feeding Progression Pattern Is Associated with Longitudinal Head-Size Growth Faltering and Neurodevelopmental Impairment Outcomes in Extremely Preterm Infants

**DOI:** 10.3390/nu15051277

**Published:** 2023-03-04

**Authors:** Yung-Chieh Lin, Chi-Hsiang Chu, Yen-Ju Chen, Ray-Bing Chen, Chao-Ching Huang

**Affiliations:** 1 Department of Pediatrics, National Cheng Kung University Hospital, College of Medicine, National Cheng Kung University, Tainan 704302, Taiwan; 2 Graduate Institute of Clinical Medicine, College of Medicine, National Cheng-Kung University, Tainan 701401, Taiwan; 3 Department of Statistics, Tunghai University, Taichung 407224, Taiwan; 4 Department of Statistics, Institute of Data Science, National Cheng Kung University, Tainan 701401, Taiwan; 5 Department of Pediatrics, College of Medicine, Taipei Medical University, Taipei 110301, Taiwan

**Keywords:** enteral feeding progression patterns, clustering analysis, growth faltering, head circumference, neurodevelopmental impairment, extremely preterm infants

## Abstract

Objective: To determine whether feeding progression patterns in the first eight postnatal weeks, depicted by clustering analysis of daily enteral feeding volume, are associated with longitudinal head-circumference (HC) growth and neurodevelopmental outcomes in extremely preterm (EP) infants. Methods: 200 infants who were admitted at gestational ages 23–27 weeks between 2011 and 2018; survived to discharge; and underwent longitudinal HC growth measurements at birth, term-equivalent age (TEA), corrected age (CA) 6-month, 12-month, and 24-month; and neurodevelopmental assessment using the Bayley Scales of Infant Development at CA 24 months were included for analysis. Results: kmlShape analysis identified two distinct enteral feeding progression patterns: fast progression in 131 (66%) infants and slow progression in 69 (34%) infants. Compared to the fast progression group, the slow progression group showed significantly lower daily enteral volumes after day 13, was older in postnatal age reaching full feeding, had a higher rate of Delta z scores of HC (zHC) < −1 (*p* < 0.001) between birth and TEA, and displayed lower longitudinal zHC from TEA to CA 24 months. The slow progression group also showed higher rates of microcephaly [42% vs. 16%, *p* < 0.001; adjusted odd ratio (aOR): 3.269, *p* = 0.001] and neurodevelopmental impairment (NDI) (38% vs. 19%, *p* = 0.007; aOR: 2.095, *p* = 0.035) at CA 24 months. For NDI, the model including feeding progression patterns showed a lower Akaike information criterion score and a better goodness of fit than the model that did not include feeding patterns. Conclusion: Characterizing feeding progression pattern may help identify EP infants at high-risk of head-size growth faltering and NDI at early childhood.

## 1. Introduction

Extremely preterm (EP) infants with gestational age <28 weeks are at high risk of adverse growth and neurodevelopmental outcomes at follow-up [1,2,3]. Poor or inadequate nutritional support is associated with long-term neurodevelopmental problems [4,5]. In early postnatal weeks, the advancement of enteral feeding in EP infants can not only be affected by gastrointestinal morbidities but also be associated with non- gastrointestinal risks, such as respiratory failure requiring mechanical ventilation [6,7]. Therefore, the enteral feeding progression pattern may be associated with the well-being and neurodevelopmental outcomes of EP infants [6,8].

Monitoring the growth trajectories of head circumference (HC) starting from birth to discharge and periodically into early childhood is critical for neurodevelopmental outcomes in EP infants [9,10,11]. Head-size growth is more related to cognitive outcomes than bodyweight (BW) because head size is more relevant to brain volume and cortical maturation before 24 months of age [12,13,14]. Poor postnatal head-size growth has been associated with impaired neurodevelopmental outcome in EP infants [10,15,16,17]. Whether the slow-feeding progression pattern is associated with inadequate longitudinal HC growth and neurodevelopmental impairment (NDI) outcome at early childhood remains unknown.

kmlShape clustering analysis, a data partitioning method, allows the grouping of individuals whose trajectories have similar forms but with shifted positions in time [18]. This study compared the feeding progression patterns in the first 56 days after birth in EP infants using kmlShape analysis, and explored the associations of different feeding progression patterns with HC changes between birth and term-equivalent age (TEA) and from TEA to corrected age (CA) 6, 12, and 24 months, as well as neurodevelopmental outcomes at CA 24 months. We hypothesized that the early-life slow-feeding progression pattern is associated with longitudinal HC growth faltering and NDI outcomes in early childhood.

## 2. Materials and Methods

### 2.1. Study Settings and Design

Among the 298 EP infants who were born and admitted with gestational age at 23–27 weeks, 226 infants survived to discharge from a tertiary university hospital from January 2011 to December 2018. 213 (94%) infants received prospective longitudinal growth follow-up assessments at TEA and CA 6, 12, and 24 months. After excluding 13 children with congenital abnormalities, genetic syndromes, or perinatal or post-discharge brain injuries, 200 children were included in the analysis (Figure S1). This study was approved by the institutional review board of the University Hospital (approval code: ER-98-135; date: 28 June 2022). Informed consent was obtained from the parents of each infant.

### 2.2. Nutritional Care Policy

Preterm infants were cared for using the similar protocol of enteral feeding and parenteral nutrition soon after birth [6,19]. HC was measured weekly, and BW was measured daily. Soon after birth, infants were administrated with 3 g/kg/day of electrolyte-free amino acids via peripheral or central intravenous routes. Once the central intravenous route was established, lipid administration was initiated at 1 g/kg/day and gradually increased to 3–4 g/kg/day. When the baby’s condition was stabilized, a tailor-made composited parenteral nutrition was prescribed. A trophic feeding volume of 10–20 mL/kg/day was started as soon as possible after birth, and maintained for 1 to 3 days or more, depending on the clinical status [20,21,22].

The daily total protein intake was controlled at 3.5–4.0 g/kg/day, and the daily total lipid intakes maintained at 3–4 g/kg/day. The glucose infusion was set at 7 g/kg/day initially and then 13–17 g/kg/day according to glucose tolerance [21,22]. Total fluid was targeted at 60–80 mL/kg in the first 24 h of life and increased with improving urine outputs. After the diuretic phase after birth, the daily fluid intake was maintained at 130 to 150 mL/kg/day with caloric density of 80–100 Kcal/kg/day.

Increase in the feeding volume by increments of 10–20 mL/kg/day was prescribed and evaluated daily. When the enteral feeding volume reached 100 mL/kg/day, feeding with fortified human milk (0.74 Kcal/mL) was initiated and intravenous lipid administration discontinued. Full enteral feeding was defined as the enteral feeding volume reaching 120 mL/kg/day [6,23,24,25] and intravenous parenteral fluids discontinued along with removal of intravenous catheters. Full enteral volume was set at 120 mL/kg/day, which is commonly used for preterm infants with balanced fluid maintenance without additional intravenous fluid support. Further milk volume advancement might be required for higher caloric requirement and growth [6,19].

The postnatal ages at initial feeding and full enteral feeding (120 mL/kg/day) were recorded. The daily enteral feeding volume in the first 8 postnatal weeks was calculated as mL/kg/day. The daily parenteral nutritional intake (mL/kg/day), the daily total nutritional fluid intake (mL/kg/day), and the daily total caloric intake (mL/kg/day) were also calculated.

After discharge, infants were regularly followed up with for health surveillance and growth and developmental supervision up to CA 24 months. Fortification of milk or the use of the post-discharge formula was shifted to the regular infant formula or unfortified human milk when the growth trajectories reached the adequate percentile [22,26].

### 2.3. Covariates

Small for gestational age (SGA) was defined as birth BW of less than the 10th percentile for GA. Neonatal morbidities were recorded, including respiratory distress syndrome (RDS) requiring surfactant therapy, hypotension requiring vasopressors, hemodynamically significant patent ductus arteriosus (hs-PDA) requiring surgery, postnatal steroid use, sepsis, intraventricular hemorrhage (IVH), cystic periventricular leukomalacia (cPVL), postnatal steroid, bacteremia, necrotizing enterocolitis (NEC), and non-NEC events requiring surgery [6]. Non-NEC events requiring surgery included meconium ileus, spontaneous intestine perforation, volvulus, and intestine adhesions [6].

### 2.4. Outcomes

The longitudinal growth of BW and HC was calculated from birth to TEA, and from TEA to CA 6, 12, and 24 months. BW and HC at TEA were recorded at the postmenstrual age of 37–42 weeks. A non-stretch tape was used and placed precisely at the broadest part of the forehead above the eyebrow, above the ears, and at the most prominent part of the occipital part of the head (https://www.cdc.gov/zika/pdfs/microcephaly_measuring.pdf, accessed on 28 February 2023). Each measurement represented a single measurement by experienced nurses. Re-measurement was required when obvious deviation from the standard growth trajectory curve was noted. The anthropometric z-scores for BW (zBW) and HC (zHC) at birth and TEA, respectively, were derived from Fenton’s postnatal growth charts [27]. A delta z score (the zBW or zHC at TEA minus the zBW or zHC at birth) of −1 or less indicated growth delay in BW or HC during hospital stay [26,28,29,30]. The zBW and zHC at each follow-up visit were based on the standards provided by the World Health Organization [31]. Microcephaly was defined as a head circumference of < 10th percentile for the age.

Neurodevelopmental outcomes at CA 24 months were assessed using the Bayley Scales of Infant Development third edition (BSID-III) [32,33]. Child psychologists who performed neurodevelopmental assessments were blinded to the early-life feeding status. The severity of cerebral palsy was measured using the Gross Motor Function Classification System (GMFCS) as mild (level 1), moderate (level 2 or 3), or severe (level 4 or 5). NDI was defined as the presence of one or more of the following: cognitive composite score or motor composite score < 85 by BSID-III, moderate or severe cerebral palsy (GMFCS level ≥2), profound visual impairment, or profound hearing loss [32].

### 2.5. Statistical Analysis

The feeding patterns were analyzed using the “kmlShape” package in R to cluster meaningful groups [6,18]. Demographics and risks were compared using chi-square or Fisher’s exact tests for categorical variables and the independent *t*-test or Mann–Whitney U test for continuous variables. Repeated-measures analysis of variance was used to compare the longitudinal anthropometric data between groups. For multiple comparisons at each time point, Bonferroni adjustment as a post hoc test was used to explore pairwise differences. A generalized estimating equation (GEE) was used to analyze the association between repeated measurements and factors. The dependence of longitudinal anthropometric variables on the risks, chosen a priori, and feeding pattern was first assessed using univariate analysis. Using logistic regression and adjusting for the risk factors selected from the candidate factors in the univariate analysis, the association between feeding patterns and NDI outcomes was analyzed. After univariate analysis, all candidate factors were included in the multivariable analysis and chosen by a stepwise procedure using the Akaike information criterion (AIC). AIC was used to identify better performance from the candidate models. The likelihood ratio test was also used to determine the goodness of fit of the two competing statistical models. Results were considered statistically significant if the *p*-value was less than 0.05.

## 3. Results

### 3.1. Risks Associated with Slow Progression Feeding Pattern

**Figure 1:**
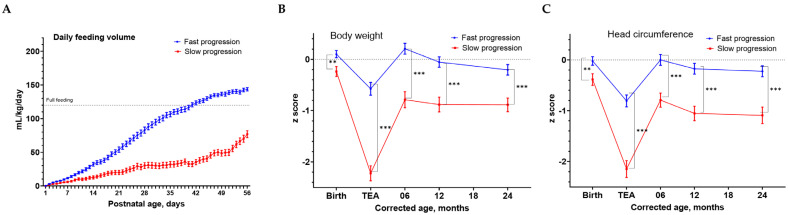
(**A**) Differences in the daily enteral feeding volumes in the first 56 postnatal days between the fast progression feeding pattern group and the slow progression feeding pattern group in extremely preterm infants. The statistical significances were observed from postnatal day 13 to day 56. Full feeding was defined as 120 mL/kg/days. Data were presented as mean ± SEM. Bonferroni adjustment was used for the multiple comparisons between the two different feeding progression patterns at each time points. Differences in the longitudinal anthropometric z-scores of bodyweights (**B**) and head circumference (**C**) from birth, term equivalent age (TEA), 6 months, 12 months, and up to 24 months of corrected age between the fast progression and slow progression feeding pattern. The blue line represents the fast progression pattern and the red line represents the slow progression pattern. Data were presented as mean ± SEM. Statistical significances: **: *p* < 0.01 and ***: *p* <0.001.

Of the 200 EP infants included for analysis, the mean gestational age was 25.6 ± 1.3 weeks, and the mean birth body weight was 839 ± 199 g. Based on the daily enteral feeding data from the first 56 postnatal days, the kmlShape analysis identified two distinct feeding progression patterns: a fast progression pattern in 131 (66%) infants (Figure S2A) and a slow progression pattern in 69 (34%) infants (Figure S2B). The slow progression pattern group was older in the postnatal ages at initial feeding and reaching full feeding and showed a significantly lower daily enteral feeding volume by postnatal day 13 (*p* < 0.05), and the differences increased up to day 56 (all *p* < 0.05) compared to the fast progression pattern group (Figure 1A).

Compared to the fast progression pattern group, the slow progression pattern group had significantly lower gestational age and birth zBW and zHC, and had higher proportions of hypotension, hs-PDA requiring surgery, late-onset sepsis, cPVL, and non-NEC gastrointestinal events requiring surgery (Table 1).

**Figure 2:**
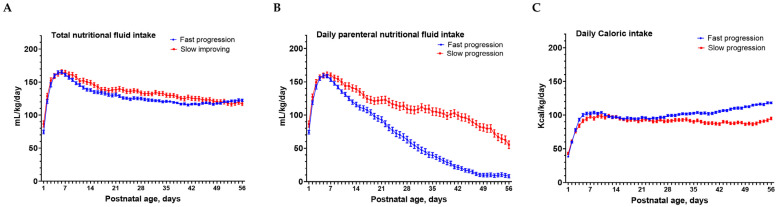
Differences of total nutritional fluid intake (mL/kg/day, (**A**)), the daily parenteral nutritional fluid intake (mL/kg/day, (**B**)), and total caloric intake (Kcal/kg/day, (**C**)) in the first 56 postnatal days between the fast progression and slow progression enteral feeding progression groups. Data were presented as mean ± SEM. Bonferroni adjustment was used for the multiple comparisons between the two feeding groups at each time point. Significant statistical differences were observed in the parenteral nutritional fluid intake from day 13 to day 56; the total nutritional fluid intake on days 26, 27, 32, and 41; the total caloric intake from day 32 to day 56.

The total nutritional fluid intakes were comparable between the two feeding progression groups except for a lower intake on days 26, 27, 32, and 41 (all *p* < 0.05) in the fast progression group (Figure 2A). The slow progression group required a longer duration on parenteral nutritional intakes from day 7 to day 56 compared to the fast progression group (Figure 2B). In contrast, the fast progression group had higher total daily caloric intakes from day 32 to day 56 (all *p* < 0.05) compared to the slow progression group (Figure 2C).

### 3.2. Slow Progression Feeding Pattern Negatively Associated with the Trend Changes of Longitudinal Head-Size Growth

The slow progression group had significantly higher rates of delta z scores < −1 in BW (86% vs. 46%, *p* < 0.001) and in HC (73% vs. 42%, *p* < 0.001) between TEA and birth than the fast progression group (Table 1). The longitudinal anthropometric zBW and zHC show that the slow progression group was associated with significantly lower zBW (Figure 1B) and zHC (Figure 1C) at TEA, and also at CA 6, 12, and 24 months compared to the fast progression group. The zHC of the fast progression group was −0.7 at TEA but increased to 0 at CA 6, 12, and 24 months. In contrast, the zHC of the slow progression group was well below −2 at TEA and remained persistently at −1 from CA 6 to 24 months.

Univariate followed by multivariate analysis by GEE for the risks associated with the trend changes of zHC showed that lower gestational age, SGA, RDS requiring surfactant therapy, severe IVH, and slow-feeding progression pattern were significantly associated with negative coefficients with repeated zHC measurements from birth to CA 24 months (Table S1).

### 3.3. Microcephaly and Neurodevelopmental Impairment Outcomes at Corrected Age 24 Months after Slow-Feeding Progression Pattern

We then compared the differences in the proportion of infants with microcephaly (HC < 10th percentile) and NDI outcomes at CA 24 months between the two early-life different feeding progression patterns. The slow progression group was associated with significantly higher rates of microcephaly [42% vs. 16%, *p* < 0.001; adjusted odd ratio (aOR): 3.269, *p* = 0.001] and NDI (38% vs. 19%, *p* = 0.007; aOR: 2.095, *p* = 0.035) than the fast progression group (Table 2).

### 3.4. The Model including Feeding Patterns Better Predicted Neurodevelopmental Outcomes

Univariate and multivariate logistic regression analyses were performed to examine early-life medical risks and feeding progression patterns associated with NDI at CA 24 months (Table 3). The model that does not include feeding progression patterns (Model 1) identified three risk factors, namely male sex (aOR 2.316, 95% CI [1.136–4.720], *p* = 0.021), lower maternal education level (2.231, [1.124–4.431], *p* = 0.022), and severe brain injury (4.331, [1.641–11.616], *p* = 0.004), as being significantly associated with NDI. After adding feeding patterns to the multivariate model (Model 2), four risk factors, that is, male sex (2.224, [1.078–4.575], *p* = 0.030), lower maternal education level (2.200, [1.098–4.406], *p* = 0.026), severe brain injury (3.854, [1.408–10.550], *p* = 0.009), and slow progression feeding pattern (2.232, [1.114–4.473], *p* = 0.024), demonstrated significantly negative impacts on neurodevelopmental outcomes. The model including feeding progression patterns showed a lower AIC score and had a better model goodness of fit using the log likelihood ratio test (*p* = 0.024) than the model that did not include feeding progression patterns.

## 4. Discussion

Under the similar enteral feeding and nutritional support protocol, we investigated the impacts of two different early-life feeding progression patterns, established by the daily enteral feeding volume in the first 8 postnatal weeks, on the longitudinal head-size growth from birth to CA 24 months, and microcephaly and neurodevelopment outcomes at CA 24 months in EP infants. We found that the slow-feeding progression pattern occurred in one third of EP infants who could be distinguished from the fast progression feeding pattern as early as postnatal day 13. The slow progression group required a longer duration on parenteral nutritional intakes from day 7, while the two feeding progression groups had similar daily caloric intakes in the first postnatal weeks. Compared to the fast progression group, the slow progression group showed growth faltering in zHC not only from birth to TEA but also from TEA to CA 24 months. The slow progression group also had significantly higher rates of microcephaly and NDI at CA 24 months. The model that included early-life feeding patterns demonstrated more negative impacts on neurodevelopmental outcomes than the model without including feeding progression patterns. Taken together, these findings suggest that close monitoring of feeding progression patterns in the early postnatal weeks provides important information on the longitudinal head-size growth and neurodevelopmental outcomes in EP infants.

### 4.1. Characterizing the Patterns of Early-Life Feeding Progression

The goal to achieve normal growth and neurodevelopment for infants born extremely preterm is to provide adequate nutritional supports through daily feeding. However, the enteral feeding progression is largely affected by the degree of gut immaturity, and the occurrence of gastrointestinal and non-gastrointestinal medical morbidities of these infants in the NICU. On the enteral feeding progression, we found that two thirds of EP infants showing the fast progression pattern had better head-size growth and neurodevelopmental outcomes compared to the slow progression pattern group. Early full enteral feeding, which appears to be feasible in these clinically stable EP infants, has been associated with better short outcome in late-onset sepsis and reduced length of hospital stays [34,35,36]. However, in these stable infants, studies fail to establish a link between increased protein and energy intakes of recommended levels and neurodevelopmental outcome at follow-up, and the evidence between early protein supplement and reduced postnatal faltering growth is weak [37,38,39,40,41]. A large, randomized trial also demonstrated a faster volume increment of enteral feeding (daily increments of 30 mL/kg) compared to a slower increment (daily increments of 18 mL/kg) failed to improve survival without NDI at CA 24 months [42,43].

Yet, very limited studies have investigated the underlying causes and growth and neurodevelopmental outcomes of the EP infants who fail to follow the trajectory of feeding progression [6,44]. We found that enteral feeding progression was very difficult to achieve in one third of EP infants due to gut immaturity and the presence of medical morbidities. The infants who followed the slow progression pattern and required prolonged parenteral nutritional intakes could be identified by the first 2 weeks after birth. Despite the comparable daily total nutritional fluid intakes and caloric intakes between the slow progression and fast progression groups in the first 4 weeks after birth, the slow progression groups showed long-term head-size growth faltering, and higher rates of microcephaly and NDI at CA 24 months.

### 4.2. Early-Life Feeding Patterns, Extrauterine Head-Size Growth by TEA, and Longitudinal Head-Size Growth from TEA to CA 24 Months

Studies on the risks for extrauterine growth restriction have included the risks, morbidities, and nutritional feeding practices in the NICU [25,45,46,47]. Head-size growth in the first two years after birth is associated with neurodevelopmental outcome in very preterm infants [10,12,13,15,16,17,48]. We focused on the daily enteral feeding volumes in the first 8 postnatal weeks to depict early-life feeding progression patterns that might associate with the extrauterine head-size growth at TEA status as well as at follow-up. Studies suggest that infants with extrauterine growth restriction at discharge/TEA frequently show continued growth restriction at follow-up [49,50,51]. Head-size growth needs to be monitored with longitudinal growth curves for the best result in EP infants [10,52,53]. We found that the slow progression feeding group was not only associated with extrauterine growth restriction in HC between TEA and birth but also with persistent head-size growth faltering from TEA to CA 24 months. Gut microbiota is important for growth in young infants [54,55]. The specific gut microbiota that were involved with the faltering of head-size changes in the slow progression feeding group remain to be elucidated.

### 4.3. Close Relationship between Early-Life Feeding Patterns, Longitudinal Head-Size Growth and Neurodevelopmental Outcomes

The relationship between growth patterns and neurodevelopment outcomes in preterm infants has been investigated [10,48,56,57]. In-hospital growth velocity in BW and HC between birth and discharge/TEA shows significant effects on neurodevelopmental outcome [12,15,48]. However, whether extrauterine growth restriction at discharge is associated with neurodevelopmental outcomes remains controversial [28,58,59]. The longitudinal growth faltering in HC from birth to TEA and into early childhood may have a higher negative predictive value for NDI than cross-sectional ones or just at TEA [10,26,28,59]. We found that slow progression feeding patterns were associated with a high rate of NDI by persistently faltering in head-size growth curves from TEA to CA 24 months. These findings indicate the close relationship between early-life feeding progression patterns, longitudinal head-size growth, and NDI outcomes in EP infants.

### 4.4. Early-Life Slow Progression Feeding Pattern Depicted by Clustering Analysis as an Important Risk for Head-Size Growth Faltering and NDI

In addition to the well-known neonatal risks that have been associated with NDI, our study demonstrated the early-life adverse feeding patterns are also an important risk for NDI. Monitoring the daily enteral feeding volume in preterm infants is a part of routine care worldwide [42]. More than 90% of infants in our NICU were fully or partially fed with human milk up to TEA [19,60,61]. kmlShape analysis stratified the heterogenic trajectories within the study populations according to their shapes after examining their time series and longitudinal data [18,62]. kmlShape clustering analysis has been applied to examine time series and longitudinal data according to their shapes in order to capture the heterogenic trajectories within the study populations. Using kmlShape clustering analysis of respiratory patterns based on the daily type of ventilation support in the first 8 weeks after birth in preterm infants, one study had shown that early-life respiratory trajectories were associated with neurodevelopmental outcomes in extremely preterm infants [32]. For the first time, we established early-life feeding progression patterns by performing clustering analysis on data from daily enteral feeding, and further linked the feeding patterns with the head-size growth trajectory and NDI in EP infants.

### 4.5. Limitations

This prospective registration and follow-up cohort of EP infants has limitations inherent to observational studies from a single academic center. The generalizability of the results to other centers needs to be validated through further prospective multicenter studies. The macronutrients, energy, and protein intakes during hospitalization may be associated with a free fat mass/fat mass at discharge, a potential marker for growth and neurodevelopment outcomes in infants [63,64]. The evaluations of macronutrients, energy, and protein intakes during hospitalization and free fat mass or fat mass at TEA and follow-up were not available in this study. A single head circumference measurement at designated time points instead of considering the average of consecutive measurements and the lack of an interobserver agreement assessment of measurements could also be study limitations.

## 5. Conclusions

Early-life feeding progression pattern may serve as a risk factor for long-term head-size growth faltering and neurodevelopmental outcomes. EP infants who followed the slow progression feeding pattern were at high risk of longitudinal head-size growth faltering, microcephaly, and NDI at early childhood. Characterizing the adverse feeding progression pattern in the first two weeks after birth may help identify high-risk infants earlier for further nutritional interventions to improve outcomes.

## Figures and Tables

**Table 1:** Differences of demographics, prenatal and neonatal risks, and exposures between the fast progression and slow progression pattern groups in extremely preterm infants.

Feeding Trajectories	Fast Progression	Slow Progression	
Risks and Exposures	N = 131	N = 69	*p* Values
**Demographics**			
Male, n (%)	64 (49)	40 (58)	0.237
Gestational age, weeks, mean (SD)	25.9 (1.3)	25.2 (1.3)	<0.001
Birth bodyweight z score, mean (SD)	0.10 (0.82)	−0.24 (0.81)	0.006
Small for gestational age, n (%)	7 (5)	6 (9)	0.377
Birth head circumference z score, mean (SD)	−0.02 (0.96)	−0.39 (0.95)	0.011
Maternal education level (<college), n (%)	50 (38)	29 (42)	0.649
**Prenatal period**			
Antenatal steroid, n (%)	123 (94)	66 (96)	0.751
Multiple gestation, n (%)	39 (30)	21 (30)	1.000
Preeclampsia, n (%)	20 (15)	12 (17)	0.690
Gestational diabetes, n (%)	4 (3)	1 (1)	0.661
**Neonatal period**			
**Respiratory/hemodynamics, n (%)**	109 (83)	62 (90)	0.291
RDS requiring surfactant therapy, n (%)	64 (49)	36 (52)	0.766
Hypotension requiring vasopressors, n (%)	82 (63)	57 (83)	0.006
hs-PDA requiring surgery, n (%)	26 (20)	28 (41)	0.002
Postnatal steroid, n (%)	26 (20)	21 (30)	0.114
**Infection events, n (%)**	17 (13)	30 (43)	0.001
Early-onset sepsis, n (%)	5 (4)	5 (7)	0.318
Late-onset sepsis, n (%)	14 (11)	26 (38)	<0.001
**Severe brain injury, n (%)**	10 (8)	11 (16)	0.089
IVH (≥III), n (%)	8 (6)	6 (9)	0.563
cPVL, n (%)	4 (3)	9 (13)	0.012
**GI morbidities, n (%)**	13 (10)	25 (36)	<0.001
NEC ≥ stage II, n (%)	10 (8)	12 (17)	0.055
Non-NEC events requiring surgery, n (%)	4 (3)	16 (23)	<0.001
**Feeding progression**			
Postnatal age at initial feeding, days, mean (SD)	4.2 (3.9)	5.9 (5.3)	0.024
Postnatal age at reaching full feeding, days, mean (SD)	33 (9.5)	56 (20.6)	<0.001
**Growth differences between TEA and birth**			
Delta z score of bodyweight <−1, n (%)	60 (46)	59 (86)	<0.001
Delta z score of head circumference <−1, n (%)	55 (42)	50 (73)	<0.001

RDS: respiratory distress syndrome; PDA: hemodynamic significant patent ductus arteriosus; NEC: Necrotizing enterocolitis; IVH: intraventricular hemorrhage; cPVL: cystic periventricular leukomalacia; TEA: term equivalent age; EUGR: extrauterine growth restriction; Delta z scores: the differences in z scores of bodyweight or head circumference between TEA and birth.

**Table 2:** Differences in proportions of microcephaly and neurodevelopmental impairment outcomes at corrected age 24 months between the fast progression and slow progression feeding pattern groups in early life.

Early-Life Feeding Trajectories	Slow Progression	Fast Progression		* Adjusted Odds Ratio (aOR)		
Outcomes	N = 69	N = 131	*p* Values	aOR	95% CI	*p* Values
Head circumference < 10th percentile, n (%)	28 (42)	21 (16)	<0.001	3.269	1.634, 6.54	0.001
Neurodevelopmental impairment, n (%)	26 (38)	25 (19)	0.007	2.095	1.052, 4.171	0.035
Moderate to severe cerebral palsy, n (%)	8 (11.6)	6 (4.6)	0.082	2.507	0.799, 7.866	0.115
Cognitive impairment, n (%)	15 (22)	19 (15)	0.252	1.462	0.663, 3.223	0.347
Profound hearing/vision impairment, n (%)	7 (10.1)	6 (4.6)	0.142	1.923	0.597, 6.193	0.273

* Adjusted with gestational age and gender.

**Table 3:** Logistic regression analysis for early-life medical risks and feeding patterns associated with neurodevelopmental impairment outcomes at corrected age 24 months.

Logistic Regression Model		Univariate				Multivariate Model 1, Without Feeding Progression Patterns				Multivariate Model 2, With Feeding Progression Patterns		
	Ref.	OR	95% CI	*p*		aOR	95% CI	*p*		aOR	95% CI	*p*
Small for gestational age	None	0.512	0.110, 2.392	0.395		---	---	---		---	---	---
Gestational age 23–25 weeks	26–27	1.868	0.983, 3.551	0.056		---	---	0.225		---	---	0.634
Male	Female	2.859	1.444, 5.661	0.003		2.316	1.136, 4.720	0.021		2.224	1.078, 4.575	0.030
Low maternal education level	(≥college)	2.099	1.101, 3.999	0.024		2.231	1.124, 4.431	0.022		2.200	1.098, 4.406	0.026
Pulmonary/hemodynamics ^a^	None	2.369	0.783, 7.171	0.127		---	---	---		---	---	---
Infection events ^b^	None	1.157	0.554, 2.418	0.698		---	---	---		---	---	---
NEC ≥ stage II	None	1.421	0.544, 3.710	0.473		---	---	---		---	---	---
Severe brain injury ^c^	None	4.786	1.88, 12.183	0.001		4.331	1.614, 11.616	0.004		3.854	1.408, 10.550	0.009
Slow progression feeding pattern	Fast	2.564	1.334, 4.928	0.005						2.232	1.114, 4.473	0.024
Model goodness of fit												
Akaike information criterion				NA		212.57						209.48
Log likelihood						204.57						199.48 *

^a^ Any of RDS requiring surfactant therapy, hypotension requiring vasopressors, hs-PDA requiring surgery, or postnatal systemic steroid; ^b^ Any of early-onset sepsis or late-onset sepsis; ^c^ Any of severe IVH or cPVL; * Log likelihood test for model goodness of fit is applied for the comparison between model 1 and model 2 (*p* = 0.024). Low maternal education level: education level below college.

## Data Availability

The corresponding author had full access to the dataset used and analyzed during the current study. The datasets used during the current study are available from the corresponding author upon reasonable request.

## Supplementary Materials

The following supporting information can be downloaded at: https://www.mdpi.com/article/10.3390/nu15051277/s1, Figure S1: A flowchart of the extremely preterm infants included for analysis; Figure S2: The kmlShape clustering analysis characterizes the feeding progression trajectories of extremely preterm infants; Table S1: The early-life medical risks and slowly feeding progression pattern that are associated with the trend changes of head circumference z scores from birth to 24 months of corrected age in extremely preterm infants: univariate and multivariate GEE analyses.
