# Supplementary material for: Early-Life Slow Enteral Feeding Progression Pattern Is Associated with Longitudinal Head-Size Growth Faltering and Neurodevelopmental Impairment Outcomes in Extremely Preterm Infants
Source: Nutrients. 2023 Mar 4;15(5):1277. doi: 10.3390/nu15051277 (PMC10005088; doi:10.3390/nu15051277)
Supplement: Supplementary file 1 [file nutrients-15-01277-s001.zip › nutrients-2231748-supplementary.pdf]

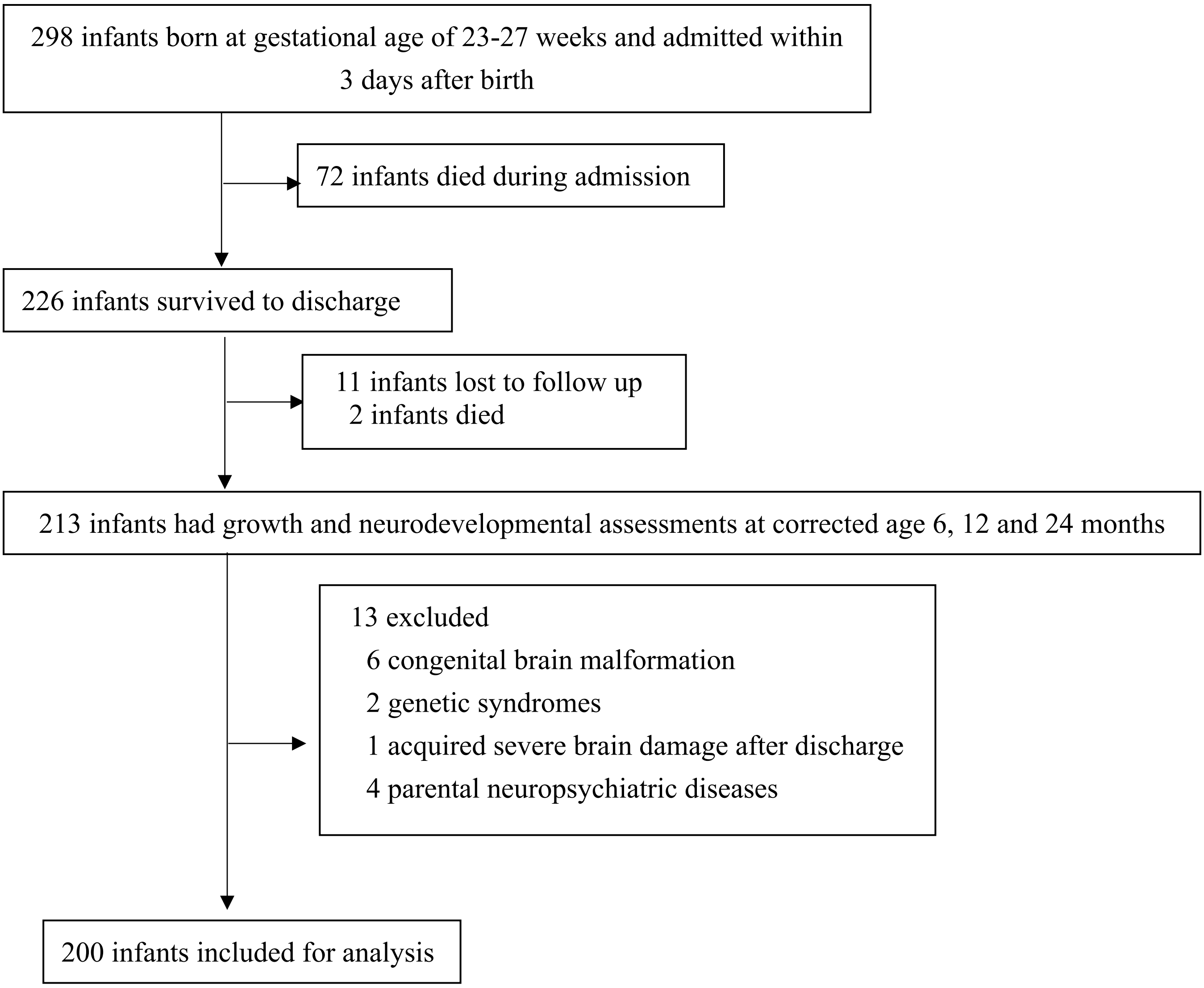

Supplemental Figure S1. A flowchart of the extremely preterm infants included for analysis.

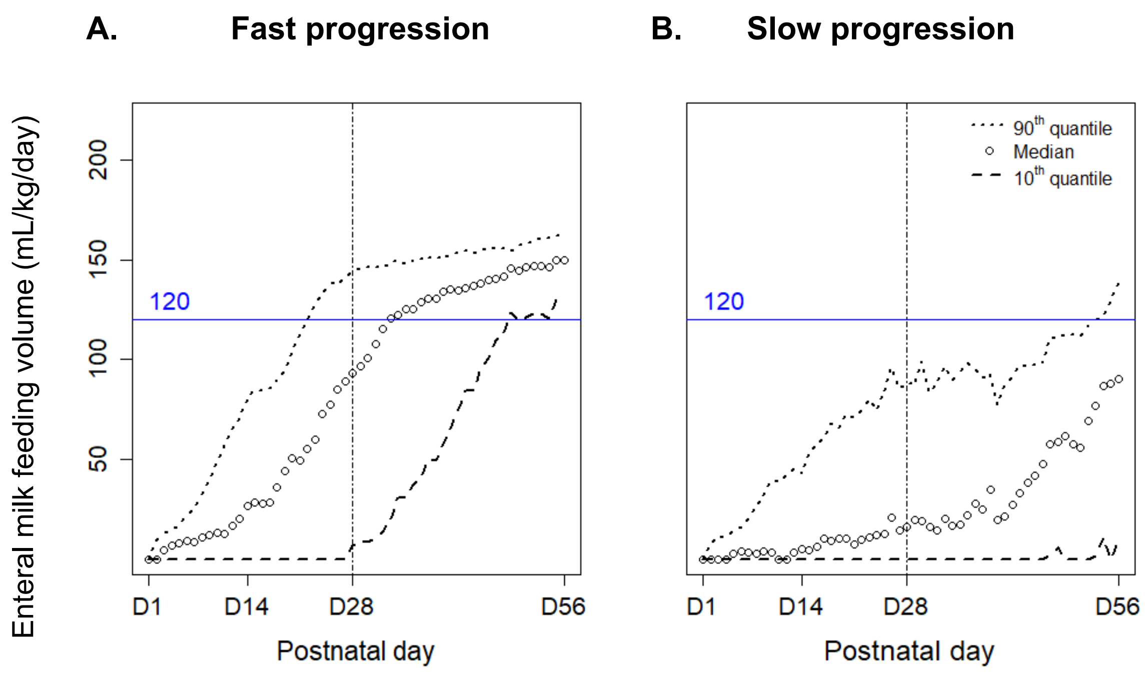

Supplemental Figure S2. The kmlShape clustering analysis characterizes the feeding progression trajectories of extremely preterm infants (N=200) based on the daily median volume of enteral milk feeding volume (mL/kg/day) in the first 56 postnatal days as fast progression pattern (n=131, **A)** and slow progression (n=69, **B**) feeding pattern. The feeding volume data were presented as the median and the 90th and 10th quantiles, and full enteral volume was defined as 120 mL/kg/day.

Supplemental Table S1. The early-life medical risks and slow feeding progression pattern that associated with the trend changes of head circumference z scores from birth to 24 months of corrected age in extremely preterm infants: univariate and multivariate of GEE analyses

	**Univariate**			**Multivariate**		
**Feeding Trajectories**	**Coefficient**	**95% CI**	***P* values**	**Coefficient**	**95% CI**	***P* values**
**Demographics**						
Gestational age, weeks	0.288	0.187, 0.388	**<0.001**	0.216	0.106, 0.325	**<0.001**
Female (ref. male)	0.189	-0.089, 0.466	0.182	0.001	-0.225, 0.226	0.994
Small for gestational age	-1.418	-1.820, -1.016	**<0.001**	-1.142	-1.577, -0.706	**<0.001**
Preeclampsia	-0.399	-0.762, -0.037	**0.031**	-0.210	-0.553, 0.133	0.231
Maternal education level (< college)	-0.057	-0.342, 0.228	0.694			
**Pulmonary/Hemodynamics**						
RDS requiring surfactant therapy	-0.593	-0.861, -0.325	**<0.001**	-0.339	-0.574, -0.104	**0.005**
**Infection events**						
Late-onset sepsis	-0.306	-0.640, 0.028	0.072	0.278	-0.017, 0.573	0.065
**Severe brain injury**	-0.985	-1.409，-0.560	**<0.001**	-0.591	-1.014, -0.168	**0.006**
**Slow feeding progression pattern, (ref. fast)**	-0.663	-0.963, -0.364	**<0.001**	-0.638	-0.884, -0.394	**<0.001**

GEE: generalized estimating equations
